# Cardiovascular magnetic resonance imaging and endomyocardial biopsy in giant cell myocarditis: a case report on diagnostic challenges and future perspectives

**DOI:** 10.1093/ehjcr/ytaf276

**Published:** 2025-06-04

**Authors:** Judith Gronwald, Karin Klingel, Andreas Schuster, Torben Lange

**Affiliations:** Department of Cardiology and Pneumology, University Medical Center of Göttingen, Georg-August University Göttingen and German Centre for Cardiovascular Research (DZHK), Partner Site Lower Saxony, Robert-Koch-Str. 40, 37075 Göttingen, Germany; Cardiopathology, Institute for Pathology and Neuropathology, University Hospital Tübingen, Liebermeisterstr. 8, 72076 Tübingen, Germany; Department of Cardiology and Pneumology, University Medical Center of Göttingen, Georg-August University Göttingen and German Centre for Cardiovascular Research (DZHK), Partner Site Lower Saxony, Robert-Koch-Str. 40, 37075 Göttingen, Germany; FORUM Medizin, An der Ziegelei 1, 37124 Rosdorf, Germany; Department of Cardiology and Pneumology, University Medical Center of Göttingen, Georg-August University Göttingen and German Centre for Cardiovascular Research (DZHK), Partner Site Lower Saxony, Robert-Koch-Str. 40, 37075 Göttingen, Germany

**Keywords:** Cardiovascular magnetic resonance, Myocarditis, Sarcoidosis, Multimodal imaging, Histopathology, Case report

## Abstract

**Background:**

Giant cell myocarditis (GCM) is a rare but often fast-progressing cardiac disease with a high risk of poor outcome. Nonetheless, its differentiation from other diseases like cardiac sarcoidosis (CS) using cardiovascular magnetic resonance imaging (CMR) remains challenging.

**Case summary:**

A 27-year-old male patient presented to the emergency department with acute cardiac decompensation and severely reduced left ventricular ejection fraction. After exclusion of an ischaemic cause of heart failure, CMR was performed, showing signs of acute inflammation and late gadolinium enhancement patterns that were indistinguishable between GCM and CS. Despite the suspicion of sarcoidosis based on a lymph node biopsy, endomyocardial biopsy (EMB) provided clear evidence of typical histopathological changes consistent with GCM. An immunosuppressive therapy was initiated leading to an improvement in left ventricular function.

**Discussion:**

Cardiovascular magnetic resonance imaging is an important cornerstone in the diagnostic pathway of GCM, however, only complementary use with EMB allows reliable diagnosis. Therefore, full diagnostic and especially prognostic potential of CMR remains unclear but offers an important starting point for optimizing patient management.

Learning points

- Differentiating giant cell myocarditis from cardiac sarcoidosis solely through cardiovascular magnetic resonance imaging remains unattainable. At present, endomyocardial biopsy is required for definite diagnosis.
- Further studies and guidelines on applying non-invasive cardiovascular magnetic resonance imaging in giant cell myocarditis for more precise diagnostics and optimized risk stratification strategies are needed.

## Introduction

Giant cell myocarditis (GCM) is a rare but severe inflammatory cardiomyopathy with an unclear aetiopathogenesis. It often progresses rapidly and carries a high risk of poor prognosis, while its clinical and imaging features closely resemble those of cardiac sarcoidosis (CS),^1^ making diagnosis particularly challenging. There is an ongoing debate on whether these conditions form a single disease continuum or represent opposite ends of a T-lymphocyte driven inflammatory cardiomyopathy.^2^ Both primarily manifest as acute heart failure (HF) and life-threatening arrhythmias caused by myocardial damage, which is at least assumed to be more pronounced in GCM.^3^ Non-invasive multimodal imaging including cardiovascular magnetic resonance imaging (CMR) and endomyocardial biopsy (EMB) are crucial for diagnosis. However, distinguishing GCM from CS remains challenging due to overlapping imaging and histopathological findings.^4^ Besides using CMR-derived T2-weighted images for depicting inflammation-caused myocardial oedema, late gadolinium enhancement (LGE) can appear ubiquitous in both conditions with distribution patterns that are indistinguishable.^1^

We present a case of acute myocardial inflammation resulting in severe heart failure, highlighting the diagnostic challenges in differentiating GCM from CS. This case underscores the potential need for future refinement of existing diagnostic and prognostic approaches in affected patients.

## Summary figure

**Figure:**
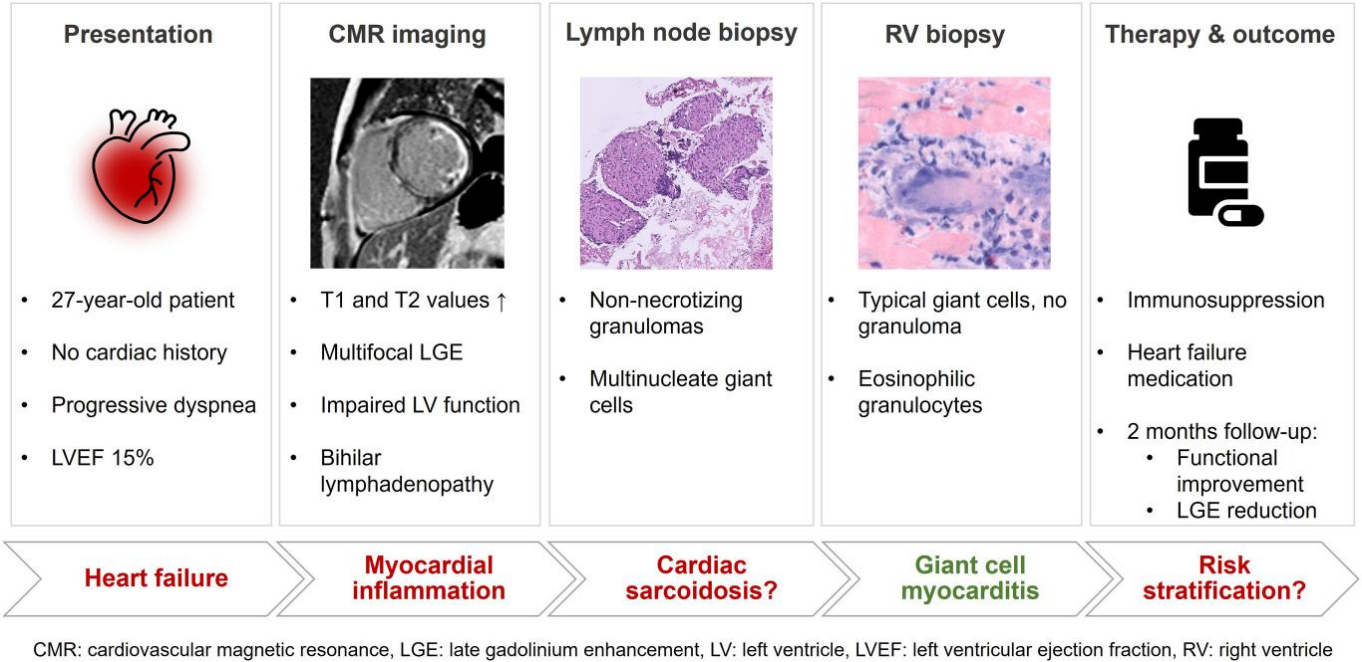


## Case summary

A 27-year-old male patient with no known history of cardiac disease presented to the emergency department with dyspnoea, increasing deterioration in general condition, fever, nausea, vomiting, and diarrhoea. The symptoms had started one week before admission. Prior to presentation, the only known pre-existing condition was biopsy-confirmed ulcerative colitis, however, no treatment had been initiated due to the absence of clinical symptoms. The family history for cardiovascular diseases was negative; smoking and illicit drug use were denied.

## History of presentation

Initial physical examination revealed signs of severe cardiac congestion. The initial mean arterial pressure was around 60 mmHg, subsequentially decreasing. Laboratory testing showed elevated troponin T (302 pg/L [<14.0]), a normal creatine kinase (66 U/L [39–308]), highly elevated NT-proBNP (31 903 pg/mL [5–125]), and elevated C-reactive protein (161 mg/L [<5]). A normal-frequency sinus rhythm with normal conduction times was documented on the electrocardiogram (ECG). On transthoracic echocardiography, a reduced left ventricular ejection fraction (LVEF) of 15% and multiple intraventricular thrombi were detected (see Supplementary material online, *Videos S1* and *S2*). Due to acute HF with progressive cardiogenic shock and subsequent onset of catecholamine dependence, the patient was transferred to the intensive care unit. Given that the clinical presentation was dominated by symptoms of acute HF and the diarrhoea at admission was neither bloody nor exceeded three episodes per day, an exacerbation of ulcerative colitis was excluded as the underlying aetiology.

## Investigations

**Figure 1:**
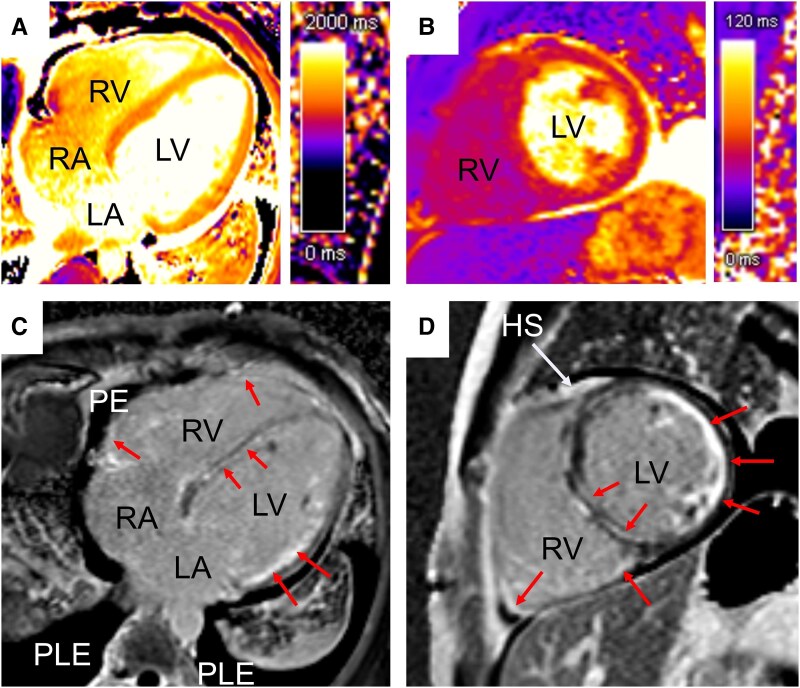
Initial cardiac magnetic resonance imaging. Cardiac magnetic resonance imaging showed ubiquitously increased T1 (*A*: T1 native: 1489 ms; ECV: 41.2%) and T2 values (*B*: 50 ms). Furthermore, multifocal late gadolinium enhancement (*C*, *D*: arrows) including the septal and lateral region as well as the anterior insertion point, extending into the right ventricular wall (*D*: ‘hook sign’), was seen in phase-sensitive inversion recovery imaging. HS, hook sign; LA, left atrium; LV, left ventricle; PE, pericardial effusion; PLE, pleural effusion; RA, right atrium; RV, right ventricle.

Differentiated catecholamine therapy with dobutamine and norepinephrine was maintained under continuous haemodynamic monitoring. Coronary artery disease was excluded by left heart catheterization, however, pronounced slow-flow within the coronary arteries was observed. Cardiovascular magnetic resonance imaging showed severely impaired global longitudinal strain (GLS) of −5% and ubiquitously increased T1 [T1 native: 1489 ms; extracellular volume (ECV): 41.2%] and T2 values (50 ms). Late gadolinium enhancement analyses (full width at half maximum technique) revealed 23% LGE of LV mass with septal-emphasized multifocal LGE and an involvement of the anterior insertion point extending across the septum into the right ventricular wall (‘hook sign’) and enhancement in the free RV wall. Additionally, subendocardial enhancement (<50% transmurality) of the entire lateral wall was detected (*Figure 1*).

**Figure 2:**
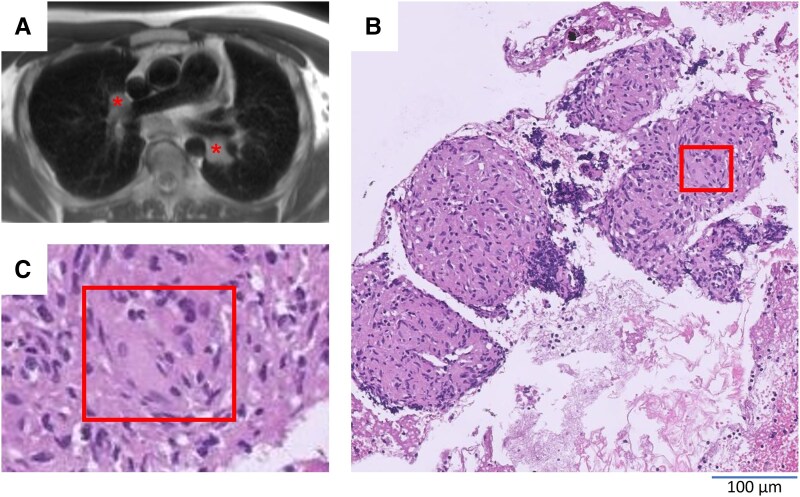
Pulmonary lymph nodes and biopsy results. Cardiovascular magnetic resonance imaging revealed bilateral hilar lymphadenopathy (*A*, asterisk). A lymph node biopsy revealed non-necrotizing granulomas (*B*) containing multinucleate giant cells (*C*), as typically seen in sarcoidosis. Histological images were kindly provided by the Institute of Pathology, University Medical Center of Goettingen, Germany.

In addition, bilateral pulmonary hilar lymphadenopathy was seen, so a bronchoscopy-guided biopsy was performed. Histopathology revealed non-necrotizing granulomas containing multinucleate giant cells; pulmonary sarcoidosis was diagnosed (*Figure 2*).

**Figure 3:**
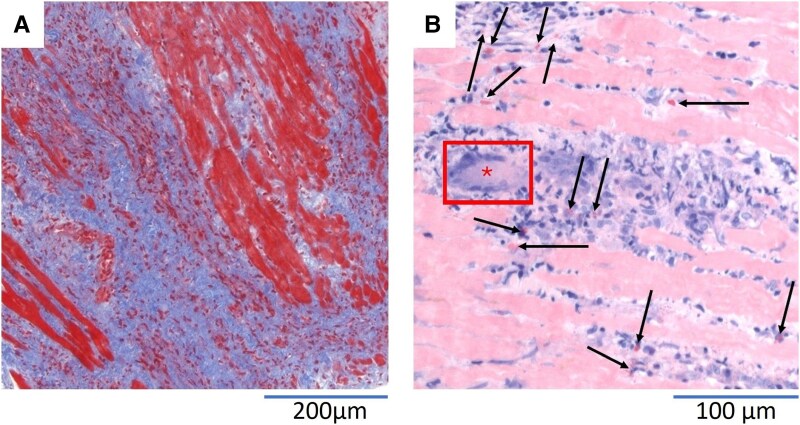
Right ventricular endomyocardial biopsy. Endomyocardial biopsy showed diffuse fibrosis and severe inflammation (*A*: blue areas; Masson trichrome). The presence of giant cells (*B*: asterisk) and increased amounts of eosinophilic granulocytes (*B*: arrows; Giemsa) was indicative of a diagnosis of giant cell myocarditis. Histological images were kindly provided by the Institute for Pathology and Neuropathology, University Hospital Tuebingen, Germany.

**Figure 4:**
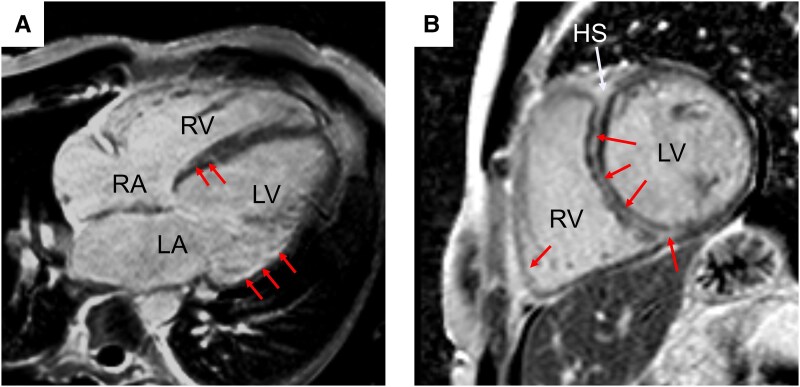
Cardiac magnetic resonance imaging eight months after symptom onset. The eight-month follow-up cardiac magnetic resonance imaging showed persistent but overall reduced late gadolinium enhancement (arrows) in phase-sensitive inversion recovery imaging. HS, hook sign; LA, left atrium; LV, left ventricle; RA, right atrium; RV, right ventricle.

Furthermore, a catheter-based endomyocardial biopsy of the RV was performed. Histological and immunohistological analysis of multiple samples revealed definite evidence of GCM, characterized by the presence of multinucleated giant cells and numerous eosinophilic granulocytes, strongly supporting the diagnosis (*Figure 3*). In contrast, there was no histopathological evidence for CS, as granulomatous formations were absent. Following current GCM guidelines, immunosuppressive therapy including prednisolone, cyclosporine, and mycophenolate mofetil was established.^5^ Following stabilization from cardiogenic shock, guideline-directed HF therapy (including sacubitril/valsartan, bisoprolol, eplerenone, and dapagliflozin) was initiated and phenprocoumon was administered due to ventricular thrombi. No relevant arrhythmias occurred. The patient received an external defibrillator (life-vest) and was discharged after six weeks in total. An eight-month follow-up CMR showed persistent LV dilatation (EDV: 154 mL/m^2^, ESV: 86 mL/m^2^) but increased GLS (−9%) and LVEF (44%). T1 (1328 ms; ECV 37.9%) and T2 (47 ms) values remained ubiquitously elevated; LGE however, showed a slight decrease to 19% of LV mass (*Figure 4*). As no thrombi were detectable anymore, phenprocoumon therapy was discontinued and the use of the life-vest was terminated due to improved LV function.

## Discussion

Giant cell myocarditis and CS share highly similar clinical presentations and CMR features, often rendering differentiation challenging. Clinically, both entities can manifest as acute HF.

Regarding CMR, both diseases can show multifocal LGE in all myocardial layers including typical imaging biomarkers like the ‘hook sign’.^4^ Subendocardial LGE, typically associated with ischaemic myocardial injury, was observed in this case. This finding may be attributed either to a globally reduced coronary vasodilator capacity—reflected by the slow-flow phenomenon seen on coronary angiography—or to the preferential subendocardial distribution of multinucleated giant cells in GCM.^6^ The latter hypothesis supports the notion that a subendocardial LGE pattern may be more indicative of GCM than of CS. However, a recent study demonstrated no significant differences in LGE distribution patterns, including the subendocardial regions, thereby most likely disproving this assumption.^1^ Thus, the primary role of CMR remains confirming widespread inflammation (and possible necrosis) in acute cases.^7,8^

In the current case, diagnosis was made more difficult by the fact that the lymph node biopsy revealed non-caseating granulomas, typical for sarcoidosis. If CS is suspected despite EMB findings supporting GCM, diagnostic algorithms include PET-CT imaging. However, cardiac 18F-fluorodeoxyglucose uptake lacks specificity to distinguish CS from other inflammatory myopathies^9^ and offers no additional clarity.

Although both GCM and CS are treated with immunomodulating agents,^5,10^ there is a clear issue on prognostic implications since transplant-free survival is known to be significantly better for patients with CS than for GCM.^11^ Furthermore, CMR-detected LGE is known to increase the risk of clinically relevant ventricular arrhythmias in these patients^11^ and is a key prognostic parameter, though data on its prognostic use in GCM remains limited. Inflammatory cardiomyopathy, as seen in both conditions, shows dynamic LGE changes, initially due to oedema, later reflecting replacement fibrosis. Persistent LGE despite oedema resolution may indicate poor prognosis, whereas ongoing LGE and oedema suggest active inflammation.^8^

Despite pronounced LGE in our patient, no malignant arrhythmias occurred during the clinical stay or outpatient period so far. This raises the question of whether an implantable cardioverter-defibrillator (ICD) implantation is necessary, when LVEF has already reached the threshold for ICD implantation of 35% and may even continue to improve.^10^ In other cardiomyopathies, the presence and extent of LGE are established indicators for ICD implantation, however, its prognostic value in GCM remains unclear.^12^ Whether an absolute LGE threshold, its distribution pattern or correlation with myocardial oedema has prognostic significance is yet to be determined.

Future endeavours might apply machine learning models for accurate diagnosis and assessing radiomics of myocardial LGE (i.e. morphologic features and textural properties), T1- and/or T2-mapping for a better distinction between GCM and CS and integrate them into risk stratification models for optimized risk prediction.^9,13^ This could enable tailored immunosuppressive therapy, adjusted indications for ICD implantation and better assessment of heart transplant urgency. Future research combining imaging and histopathology is needed to clarify the role of CMR in GCM and to enhance our understanding of the disease.

## Conclusion

The use of CMR in the diagnosis and differentiation of GCM from other diseases, such as CS, remains challenging. Specifically, the diagnostic and prognostic potential of CMR has not been fully utilized. Future studies may build upon this to better define non-invasive imaging markers of GCM, thereby reducing diagnostic uncertainties and enhancing risk stratification in affected patients.

## Lead author biography


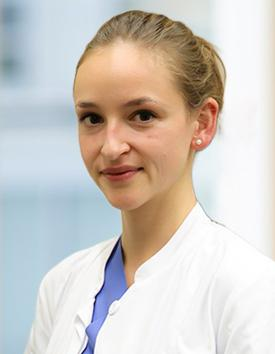


Judith Gronwald is a third-year resident at the Department of Cardiology and Pneumology at the University Medical Center of Goettingen, Germany with a special interest in cardiovascular imaging.

## Supplementary Material

ytaf276_Supplementary_Data

## Data Availability

The data underlying this article will be shared on reasonable request to the corresponding author.

## Notes

**Consent:** The authors confirm that a written consent for submission and publication of this case report including images and text was obtained from the patient in line with COPE guidance.

**Funding:** We acknowledge support by the Open Access Publication Funds of the Goettingen University.
